# Supplementary material for: The Natural Oligoribonucleotides Functionalized by D-Mannitol Affected Interactions of Hemagglutinin with Glycan Receptor Indicating Anti-Influenza Activity
Source: Membranes (Basel). 2021 Sep 30;11(10):757. doi: 10.3390/membranes11100757 (PMC8538433; doi:10.3390/membranes11100757)
Supplement: Supplementary file 1 [file membranes-11-00757-s001.zip › membranes-1344226-supplementary.pdf]

Supplementary Materials:

The natural oligoribonucleotides functionalized by D-mannitol affected interactions of human hemagglutinin with glycan receptor indicating anti-influenza activity

Zenoviy Tkachuk, Nataliia Melnichuk, Roman O. Nikolaiev, Kosma Szutkowski, and Igor Zhukov

Table S1: Binding energies and dissociation constants evaluated for the complexes with of HA3 proten with A3-ORNs and A3-ORNs-D-M in obtained by docking procedures by the Yasara software

**Substrate**	ORNs		ORNs-D-M	
	**Binding energy kcal/mol**	**Dissociation constant nM**	**Binding energy kcal/mol**	**Dissociation constant nM**
A3-ORNs	10.95	9.4	8.81	347.3
	10.95	9.4	8.81	347.3
	10.75	13.1	8.78	366.6
	10.75	13.1	8.78	366.6
	10.65	15.6	8.37	736.1
	10.65	15.6	8.37	736.1
	10.48	20.8	7.90	1627.1
	10.48	20.8	7.90	1627.1
	10.20	33.1	7.82	1859.2
	10.20	33.1	7.82	1859.2
	10.15	36.1	7.69	2303.7
	10.15	36.1	7.69	2303.7
	10.13	37.5	7.63	2536.3
	10.13	37.5	7.63	2536.3
	10.00	47.0	7.63	2570.8
	10.00	47.0	7.63	2570.8
	9.94	52.0	7.59	2745.7
	9.94	52.0	7.59	2745.7
	9.84	61.6	7.57	2816.1
	9.84	61.6	7.57	2816.1
	9.73	74.3	7.53	3038.3
	9.73	74.3	7.53	3038.3
	9.64	86.4	7.47	3356.5
	9.64	86.4	7.47	3356.5
	9.58	94.5	7.46	3396.4
	9.57	97.3	7.46	3407.9
	9.30	153.2	7.45	3477.6
	9.15	195.0	7.40	3752.0
	9.08	219.8	7.40	3777.4
	8.97	264.2	7.38	3880.8
	8.94	282.2	7.38	3880.8
	8.92	289.9	7.37	3940.2

Table S2: Binding energies and dissociation constants evaluated for the complexes with of HA3 proten with A6-ORNs and A6-ORNs-D-M in obtained by docking procedures by the Yasara software

Substrate	ORNs		ORNs-D-M	
	**Binding energy kcal/mol**	**Dissociation constant nM**	**Binding energy kcal/mol**	**Dissociation constant nM**
A6-ORNs	10.75	13.2	7.25	4849.3
	10.75	13.2	7.25	4849.3
	10.40	23.7	7.21	5161.8
	10.40	23.7	7.21	5161.8
	10.32	27.3	7.09	6310.0
	10.32	27.3	7.09	6310.0
	10.24	31.0	7.00	7432.4
	10.24	31.0	7.00	7432.4
	10.24	31.2	6.93	8350.4
	10.24	31.2	6.93	8350.4
	10.23	31.8	6.74	11410.8
	10.23	31.8	6.74	11410.8
	10.11	39.0	6.71	12064.4
	10.11	39.0	6.71	12064.4
	9.99	47.5	6.53	16374.9
	9.99	47.5	6.53	16374.9
	9.97	49.0	6.42	19748.9
	9.97	49.0	6.42	19748.9
	9.95	50.7	6.27	25267.5
	9.95	50.7	6.27	25267.5
	9.81	64.6	6.22	27632.0
	9.81	64.6	6.22	27632.0
	9.59	92.8	5.97	42351.0
	9.59	92.8	5.97	42351.0
	9.54	100.8	5.93	45004.1
	9.54	101.6	5.90	47662.3
	9.48	111.5	5.80	55951.4
	9.45	119.3	5.59	80157.0
	9.38	132.3	5.56	84178.1
	9.37	135.4	5.56	84462.7
	9.31	150.6	5.54	87657.9
	9.26	163.3	5.52	90057.4

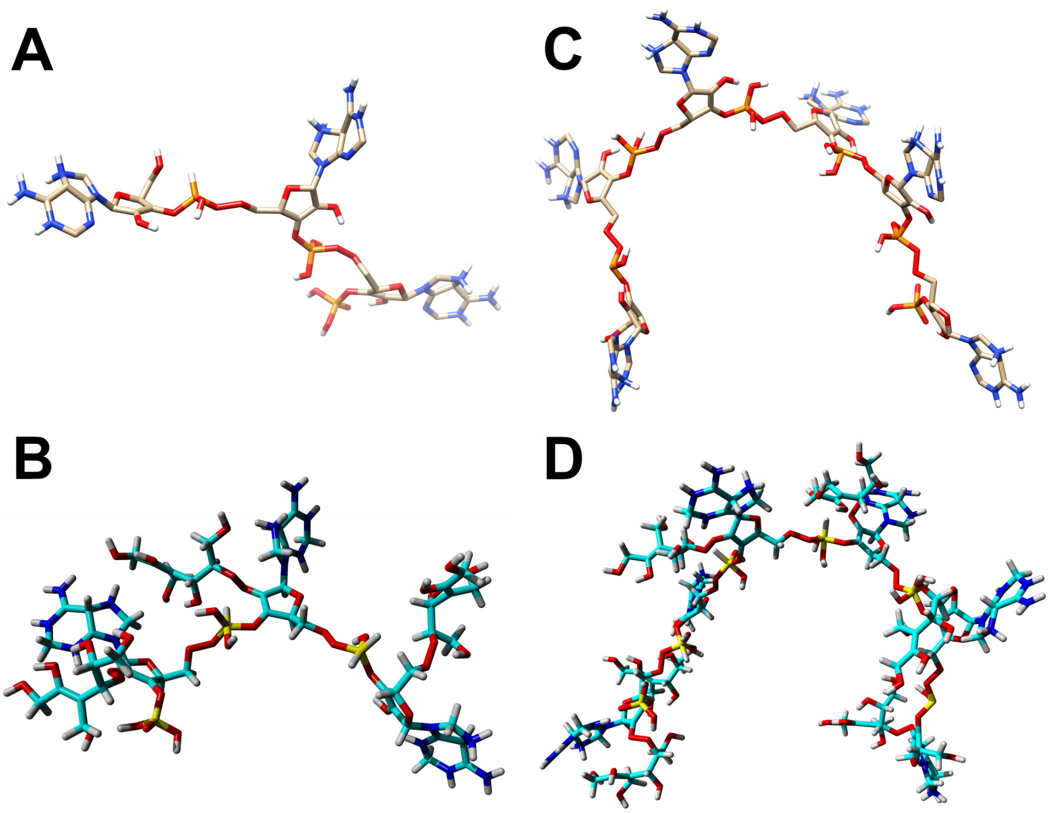

Figure S1. The structure of ligands used for docking in AutoDock Vina procedure. **(A)** A3-ORNs; **(B)** A3-ORNs-D-M; **(C)** A6-ORNs; **(D)** A6-ORNs-D-M.

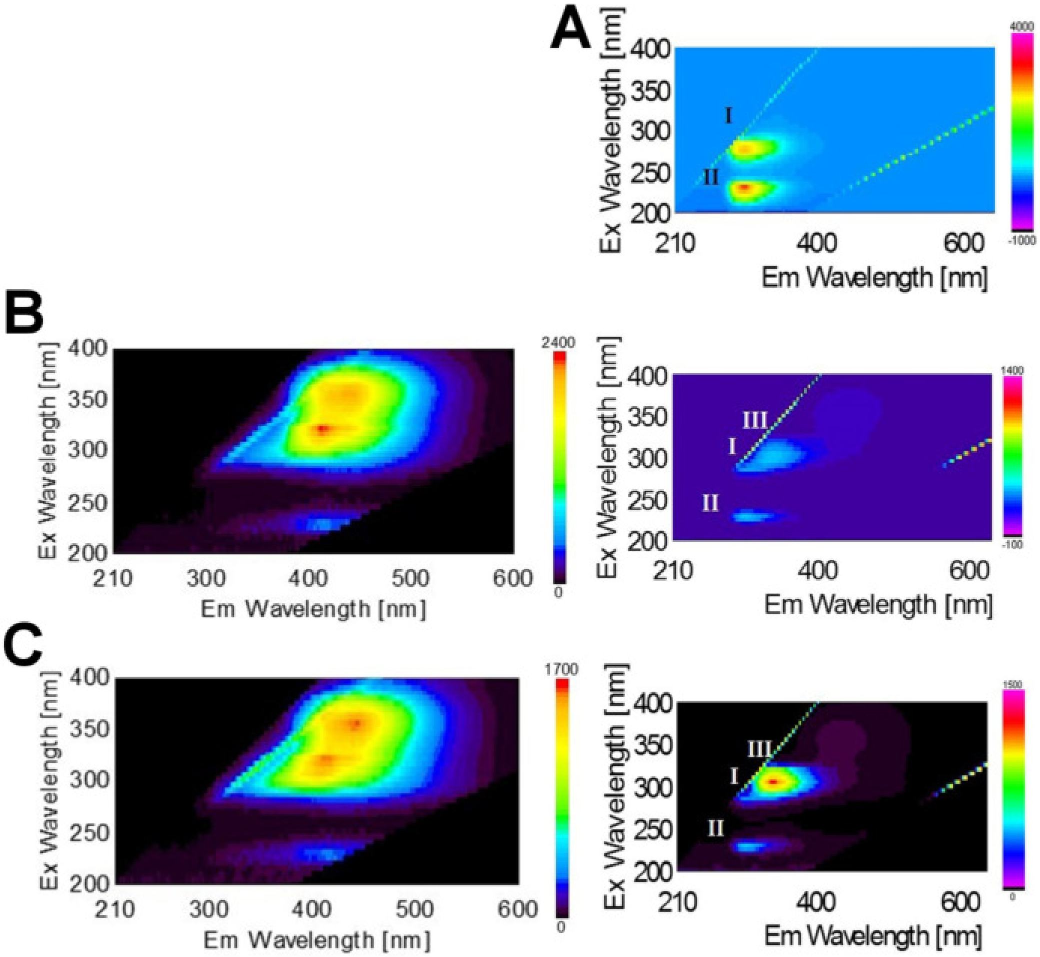

Figure S2. The total fluorescence data obtained for **(A)** – HA, **(B)** – ORNs and HA-ORNs, **(C)** – ORNs-D-M and HA-ORNs-D-M. Data for substrates (ORNs and ORNs-D-M) and complexes (HA-ONs and ORNs-D-M) are shown on panels **(B)** and **(C)** on left and right, respectively.

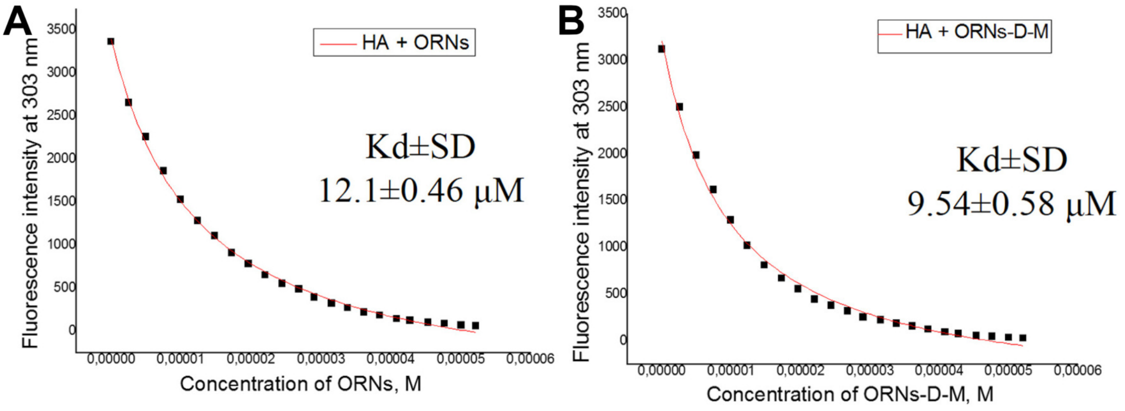

Figure S3. Dependence of fluorescence intensity on concentration of the ORNs (**A**) and ORNs-D-M (**B**).

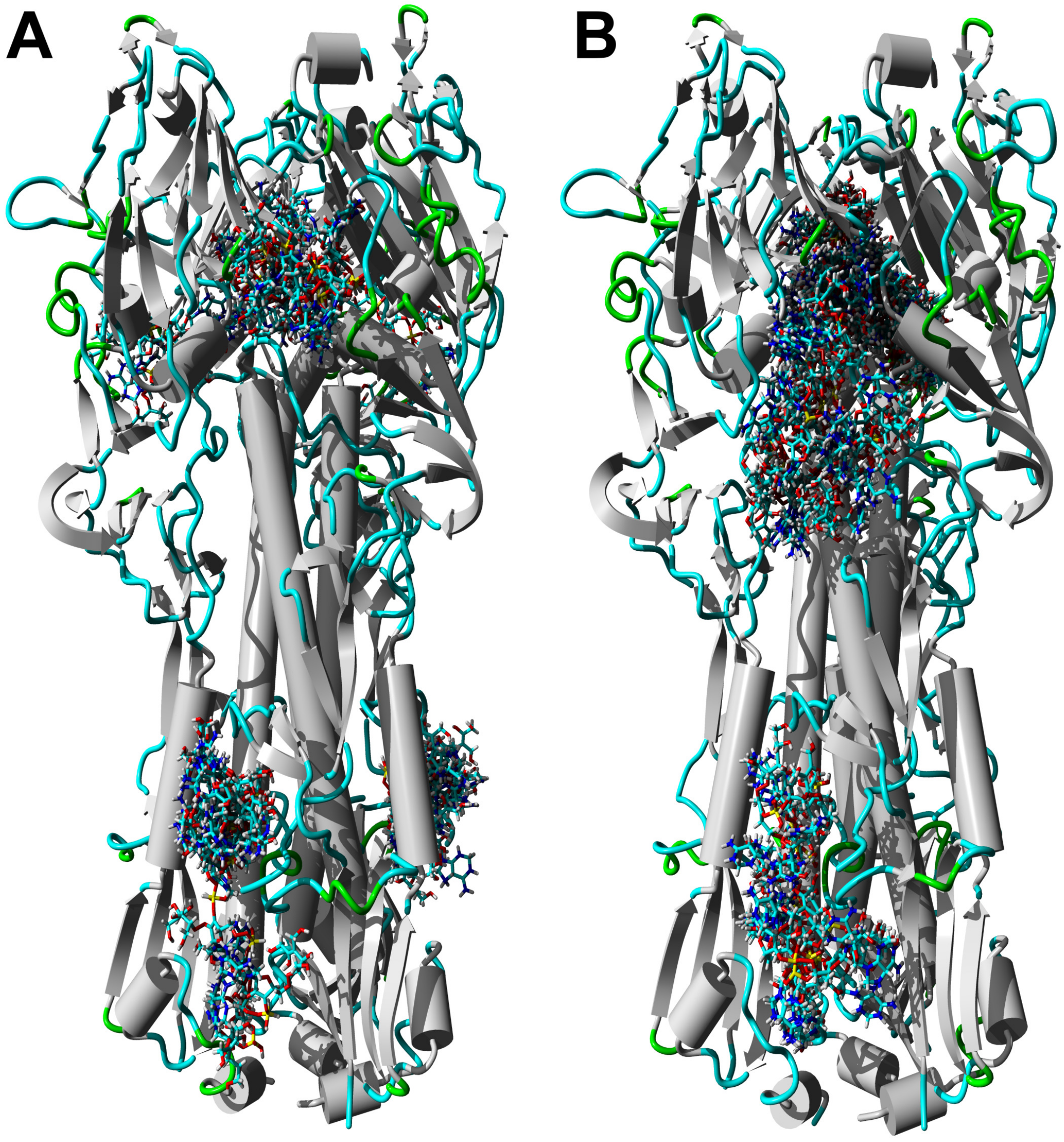

Figure S4. The docking simulations performed with AutoDock Vina program includer in Yasara software bundle. **A**) 3D structure of complex HA3 with A3-ORNs-D-M. **B)** Complex of the HA3 hemagglutinin with 32 substrates A6-ORNs-D-M.

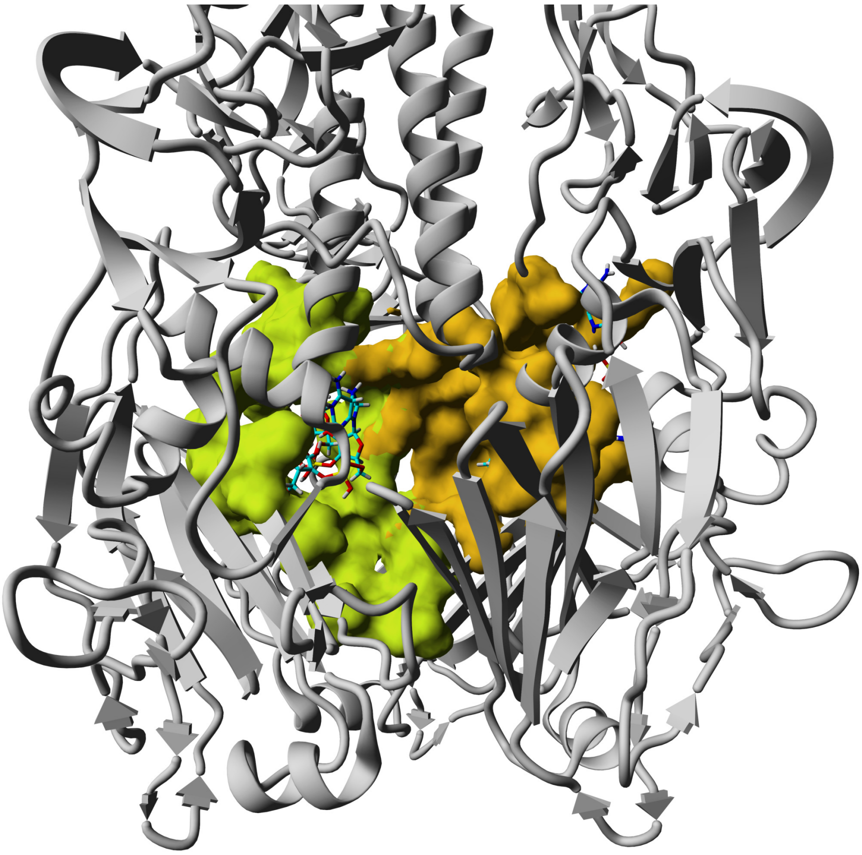

Figure S5. The structure of HA3 complex with two A3-ORNs ligands inside the RBD cavity evaluated with AutoDock Vina procedure.

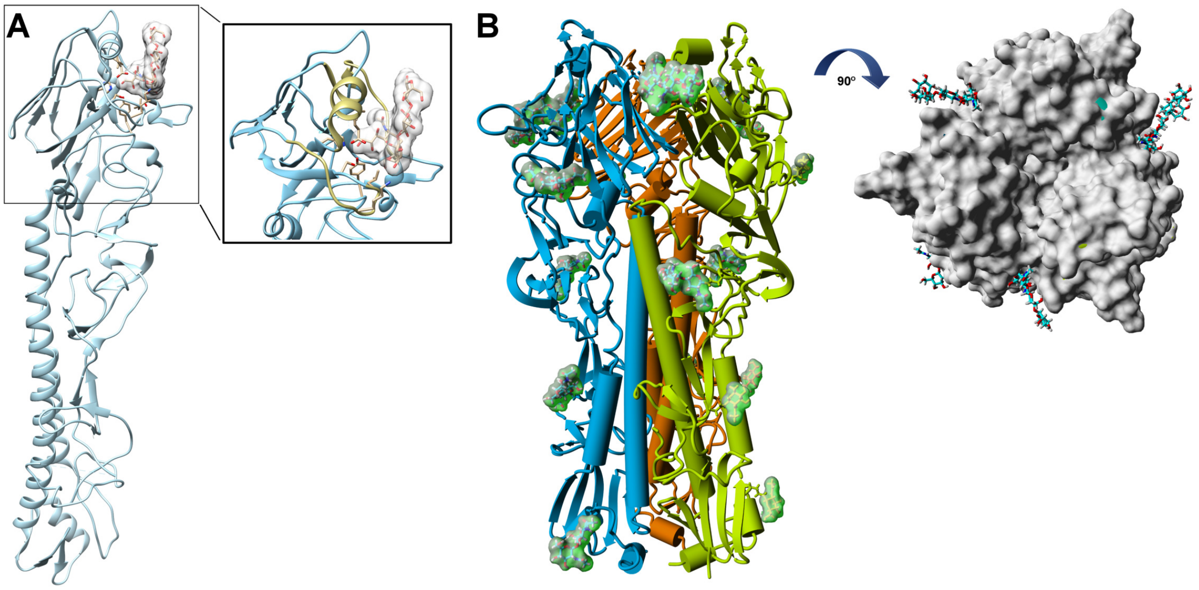

Figure S6. 3D structure of the HA protein from the different strains of the influenza virus. **(A)** One subunit (HA_1 and HA_2 chains) of the HA trimer for H2N2 pandemic variant A/Singapure/1957 (pdb 2wr7). Inset presented the sialopentasaccharide analogues of human receptor. The conserved epitops formed sialic acid binding site comprise central α-helix (residues 190 – 199) together with two loops (residues 130 – 140 and 220 – 230), highlighted as green. **(B)** Structure of HA trimer from pandemic (A/HONG KONG/1/1968) variant H3N2 influenza virus (pdb 5t6n). Linked derivatives of the sialic acid are presented demonstrated importance of glycosylation hemagglutinin for proper binding to the glycan receptor. On the right, the 3D structure of the HA3 protein is rotated, the RBD fragment is presented as gray surface with glycans located at the distal end of the viral membrane are highlighted.

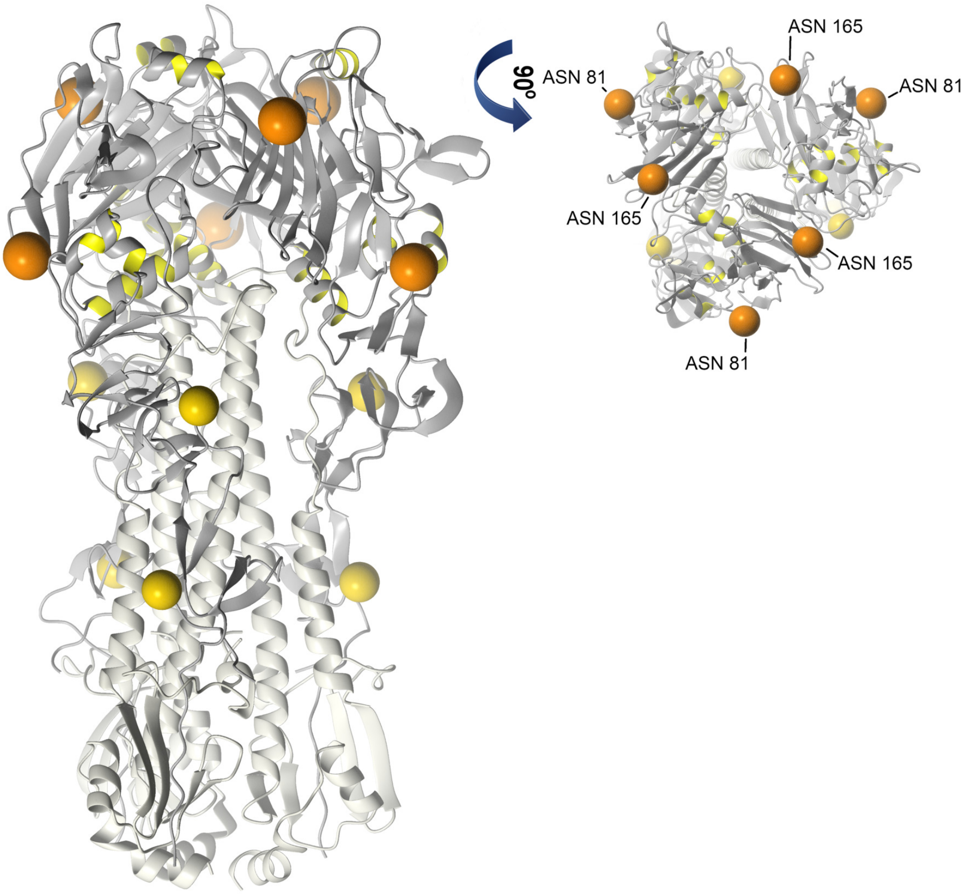

Figure S7. 3D structure of the HA protein from the H3N2 (A/HONG KONG/1/1968) virus (pdb 5t6n). Positions of the N-glycosylation sites are shown as balls and numbering of the Asn residues. Glycosylation positions located at the distal end of the viral membrane (Asn81 and Asn165) are highlighted as orange.

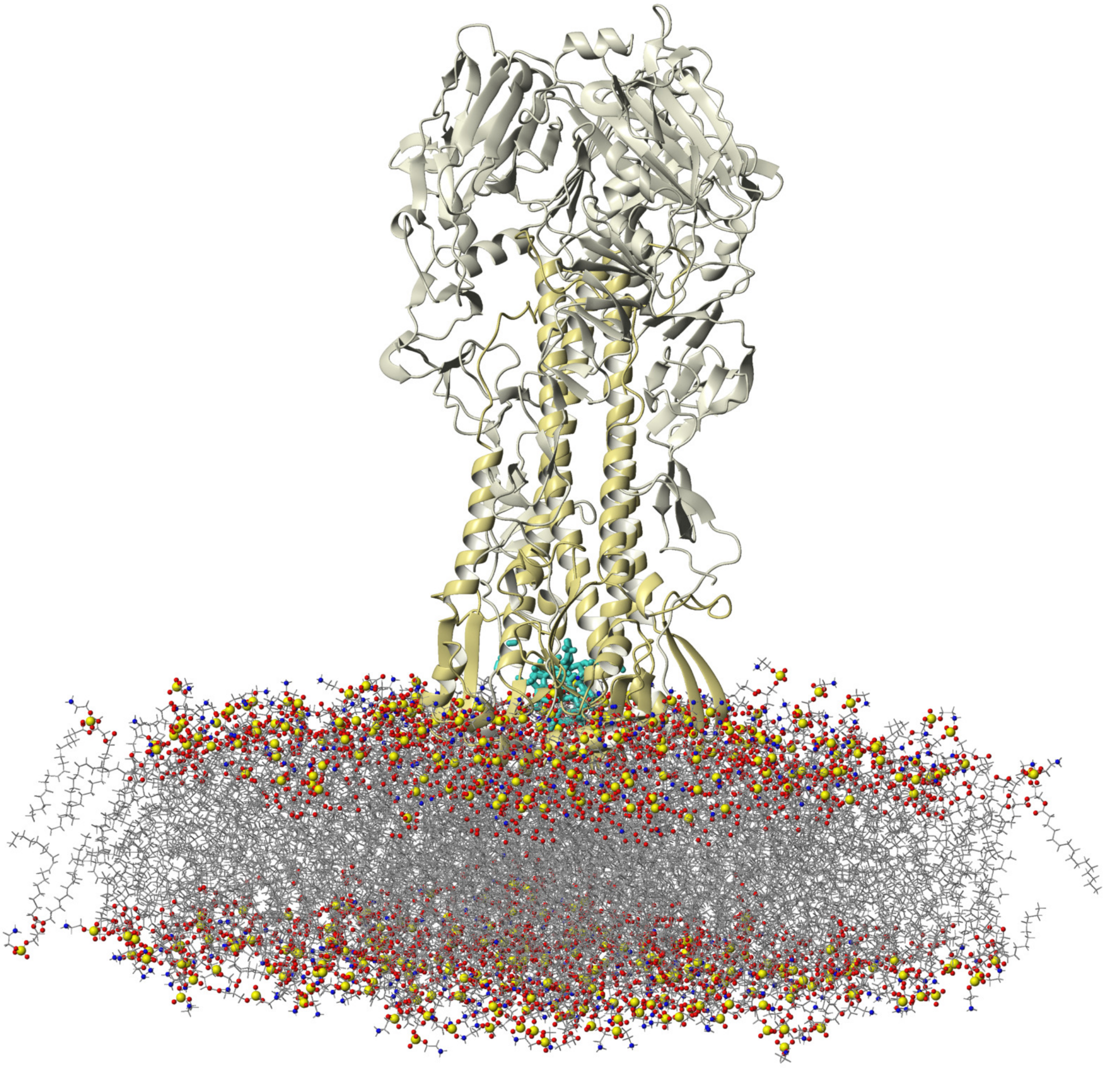

Figure S8. Proposed scheme of the interaction complex of the human HA3 hemagglutinin with A3-ORNs bound to the HA_2 subunit. As a model of the cell membrane, we use the phosphatidylethanolamine lipid created by Yasara software.
