# Supplementary figures and images for: The Natural Oligoribonucleotides Functionalized by D-Mannitol Affected Interactions of Hemagglutinin with Glycan Receptor Indicating Anti-Influenza Activity
Source: Membranes (Basel). 2021 Sep 30;11(10):757. doi: 10.3390/membranes11100757 (PMC8538433; doi:10.3390/membranes11100757)

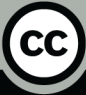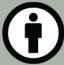

BY

Supplement: Supplementary file 1 [file membranes-11-00757-s001.zip › Definitions/logo-ccby-eps-converted-to.pdf]

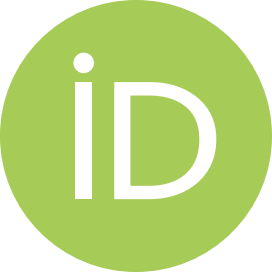

Supplement: Supplementary file 1 [file membranes-11-00757-s001.zip › Definitions/logo-orcid-eps-converted-to.pdf]

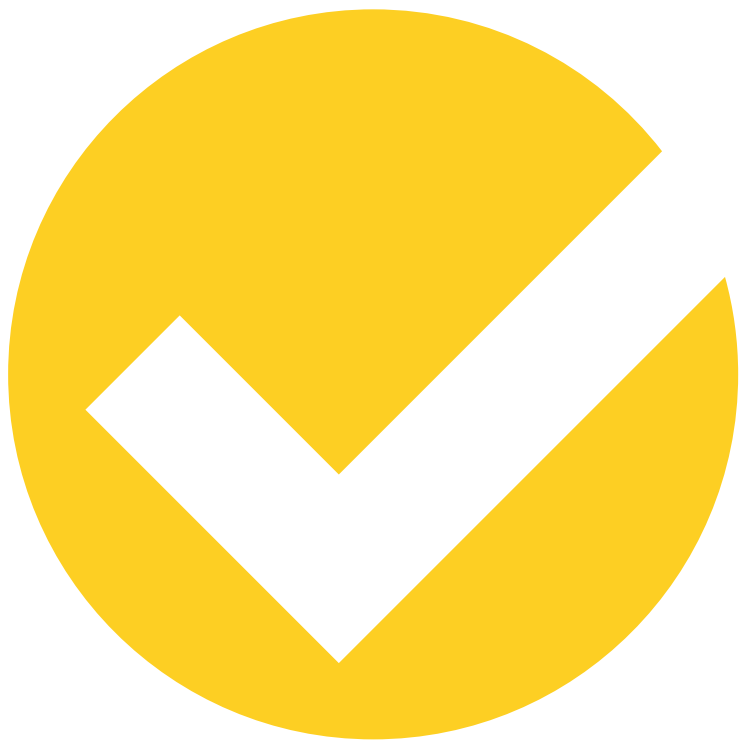

check for  
updates

Supplement: Supplementary file 1 [file membranes-11-00757-s001.zip › Definitions/logo-updates.pdf]

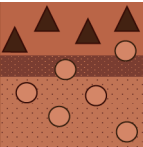

*membranes*

Supplement: Supplementary file 1 [file membranes-11-00757-s001.zip › Definitions/membranes-logo-eps-converted-to.pdf]

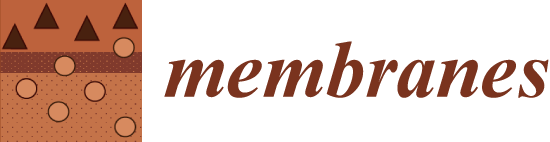

Supplement: Supplementary file 1 [file membranes-11-00757-s001.zip › Definitions/membranes-logo.png]

**A**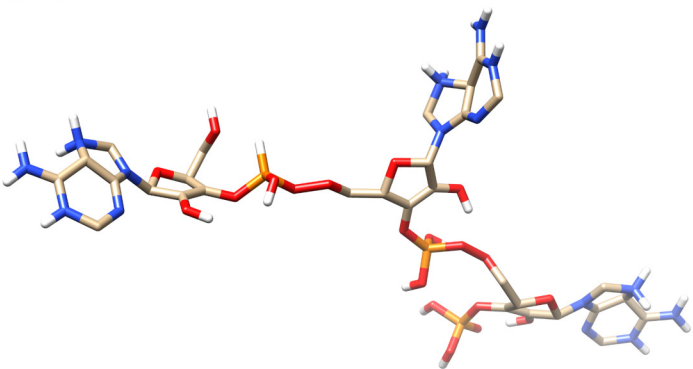**C**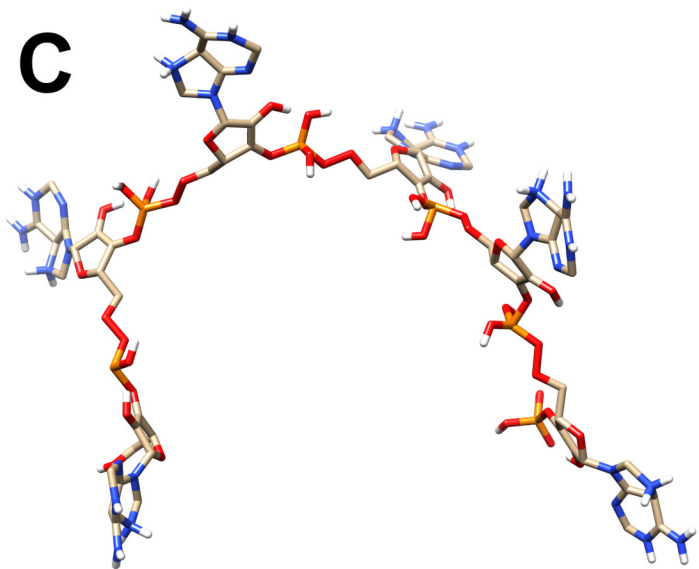**B**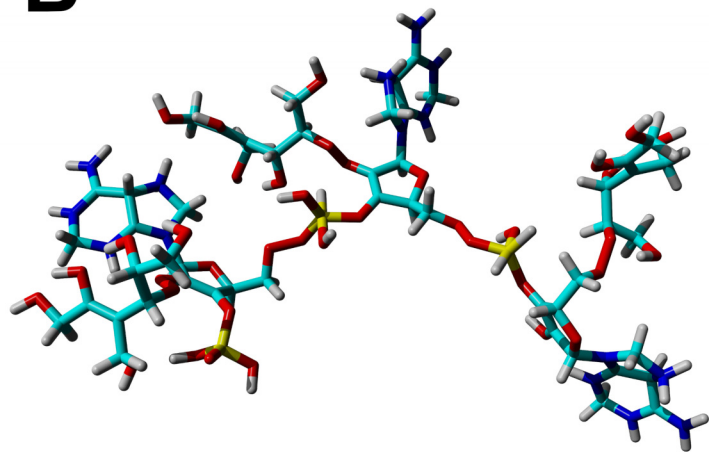**D**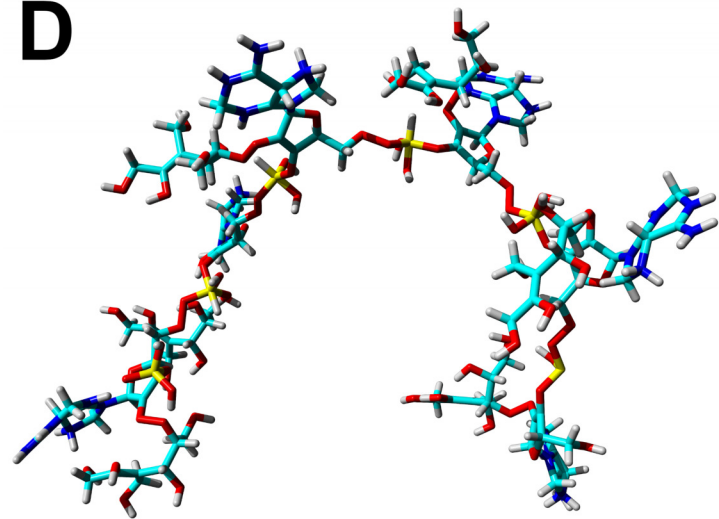

Supplement: Supplementary file 1 [file membranes-11-00757-s001.zip › SupportingMaterials/A3-A6-ORNs-eps-converted-to.pdf]

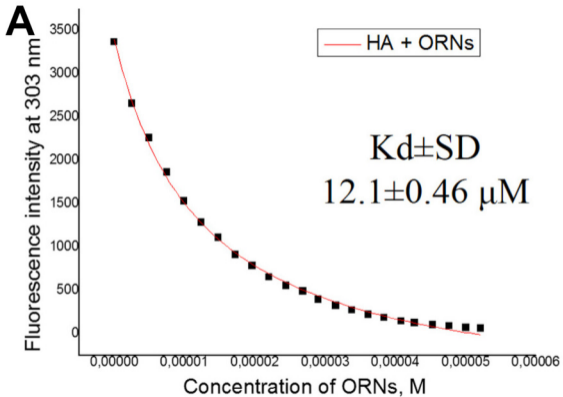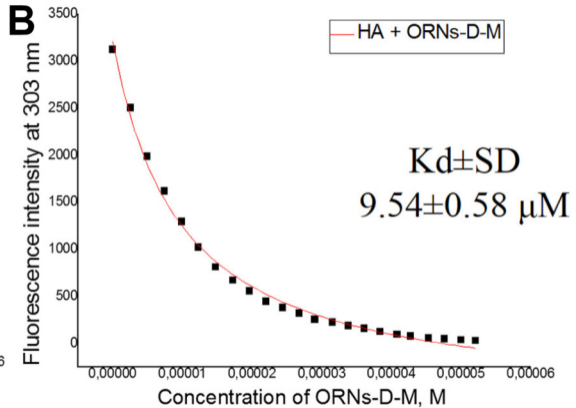

Supplement: Supplementary file 1 [file membranes-11-00757-s001.zip › SupportingMaterials/fit_fluorescence-eps-converted-to.pdf]

**A**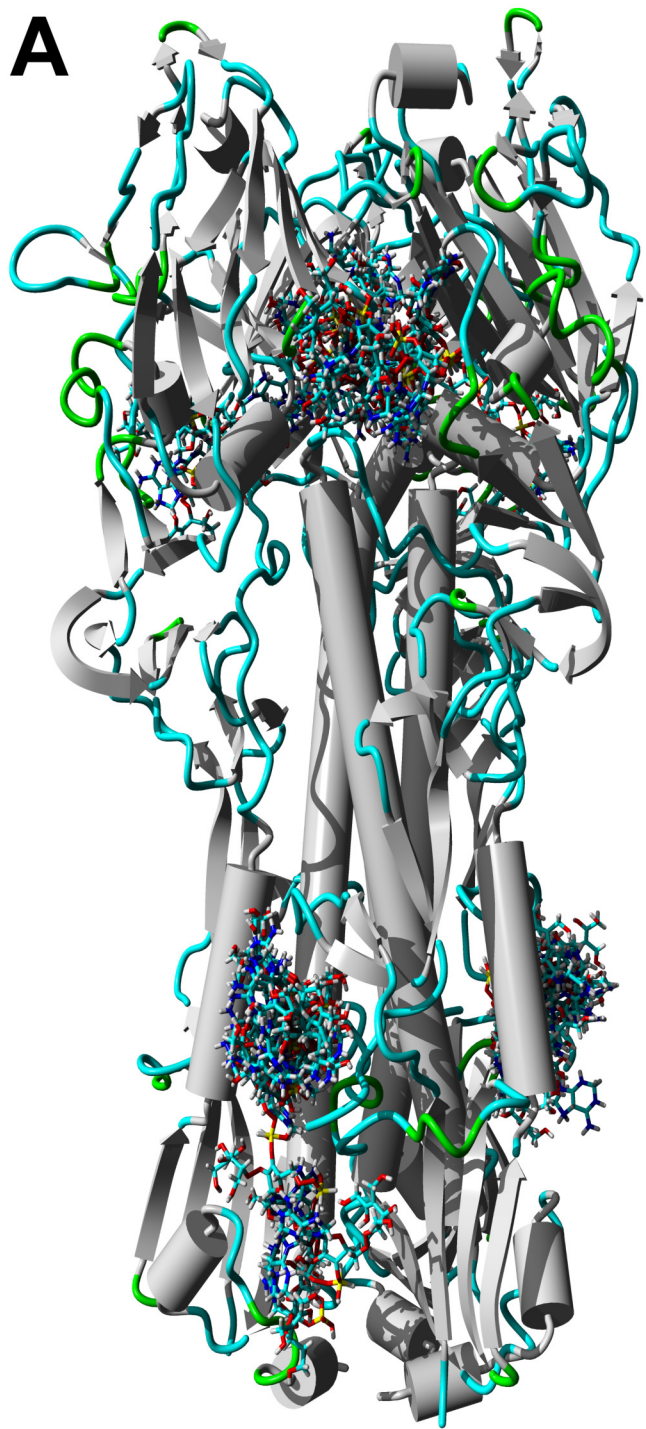**B**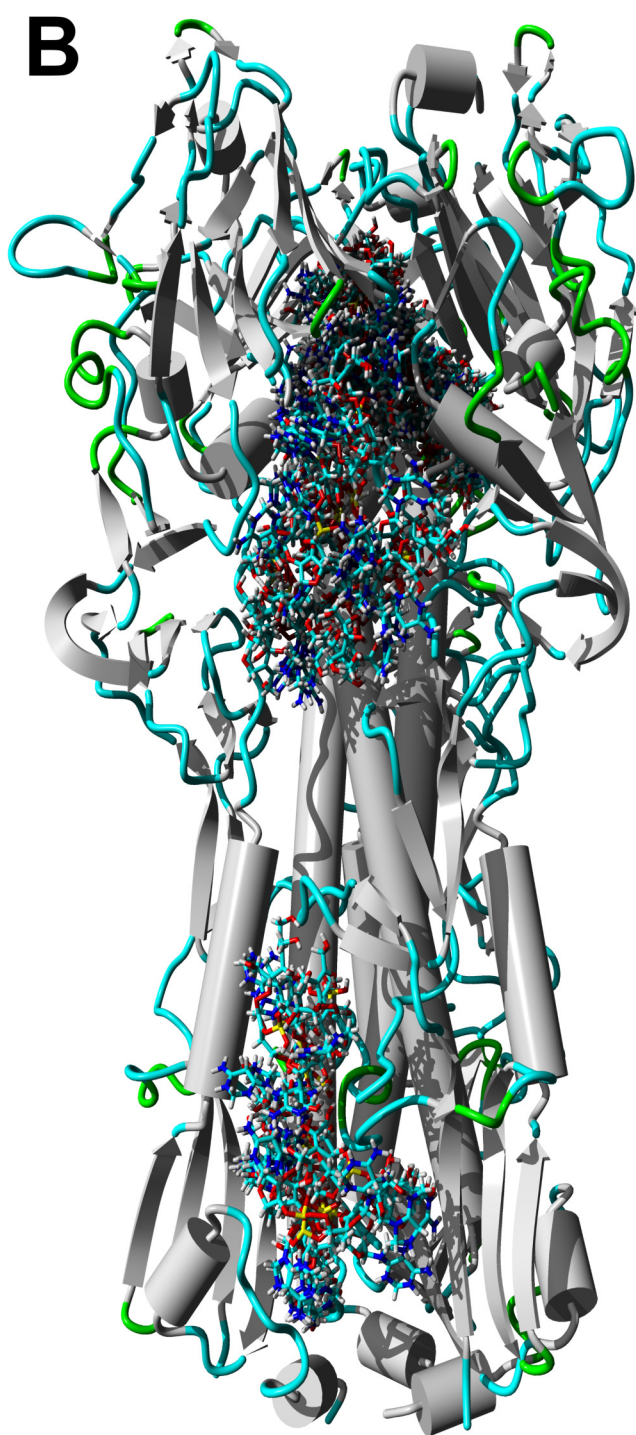

Supplement: Supplementary file 1 [file membranes-11-00757-s001.zip › SupportingMaterials/ha3_a3_a6_orn_dm-eps-converted-to.pdf]

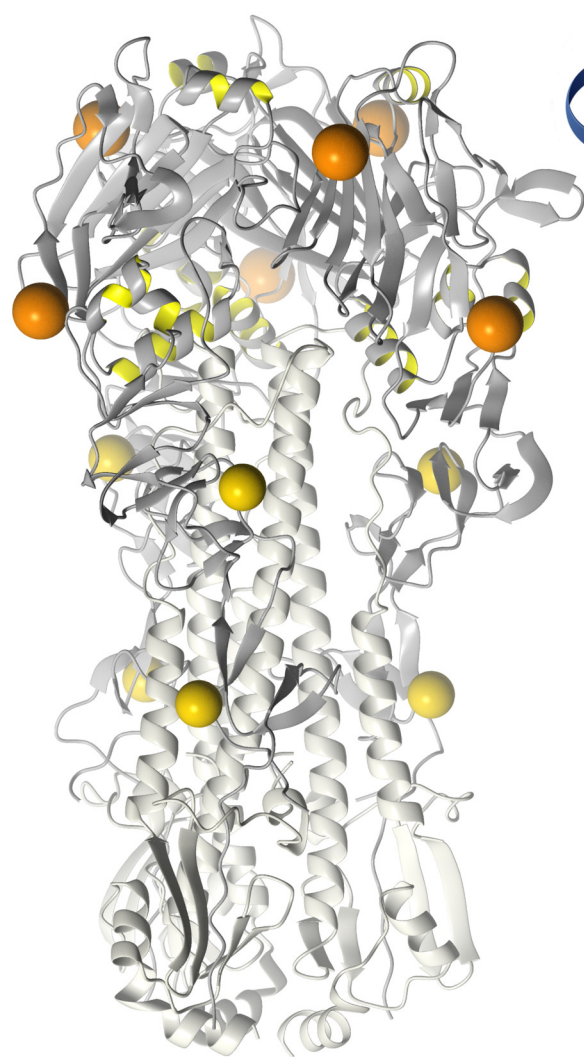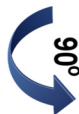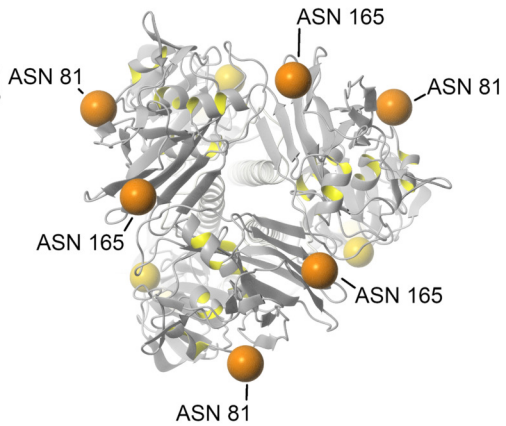

Supplement: Supplementary file 1 [file membranes-11-00757-s001.zip › SupportingMaterials/ha_glycosylation-eps-converted-to.pdf]

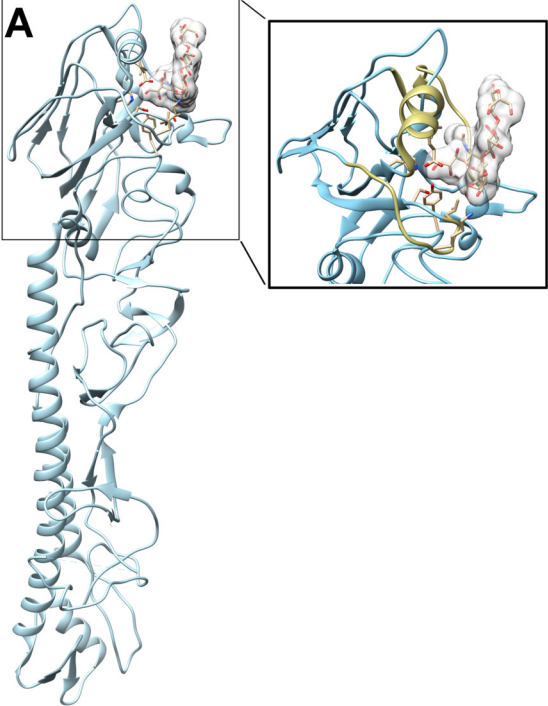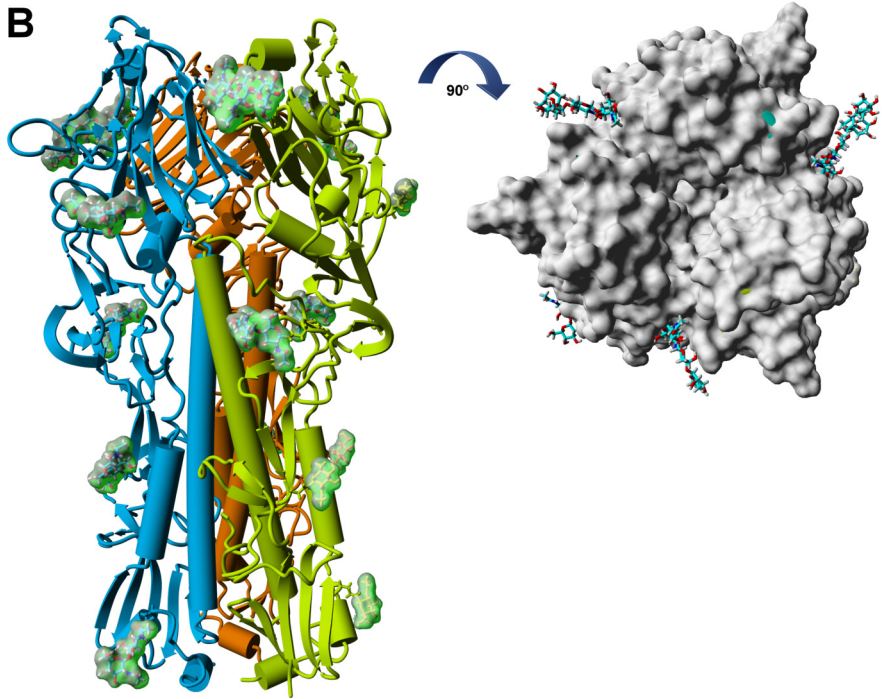

Supplement: Supplementary file 1 [file membranes-11-00757-s001.zip › SupportingMaterials/ha_models-eps-converted-to.pdf]

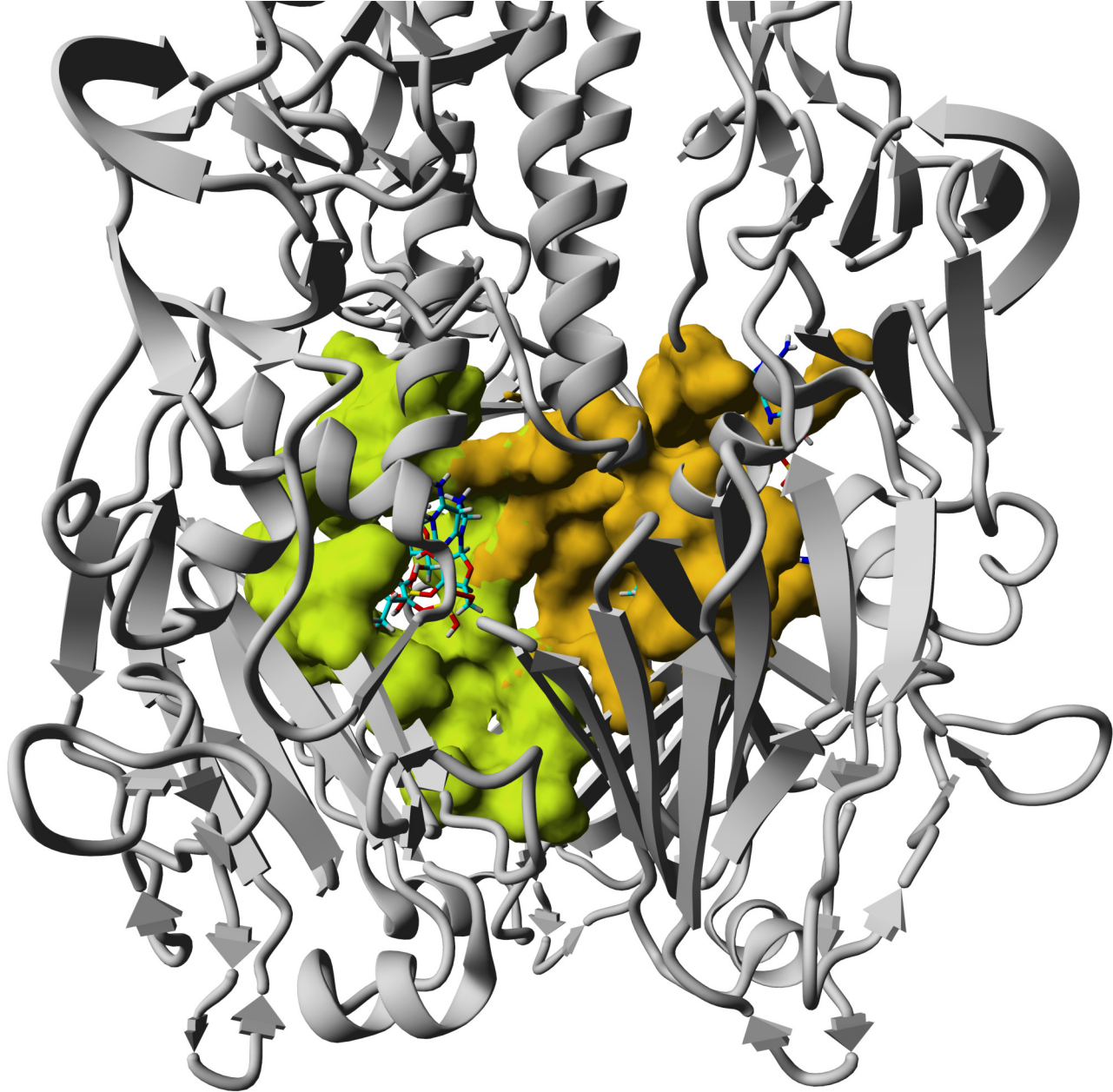

Supplement: Supplementary file 1 [file membranes-11-00757-s001.zip › SupportingMaterials/ha_ORN_A3_twosub-eps-converted-to.pdf]

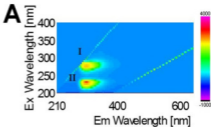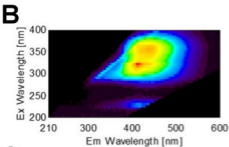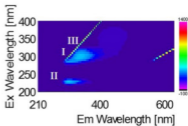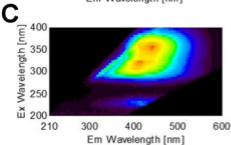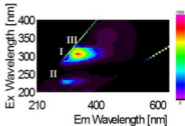

Supplement: Supplementary file 1 [file membranes-11-00757-s001.zip › SupportingMaterials/ha_orn_dm_fluorescence-eps-converted-to.pdf]

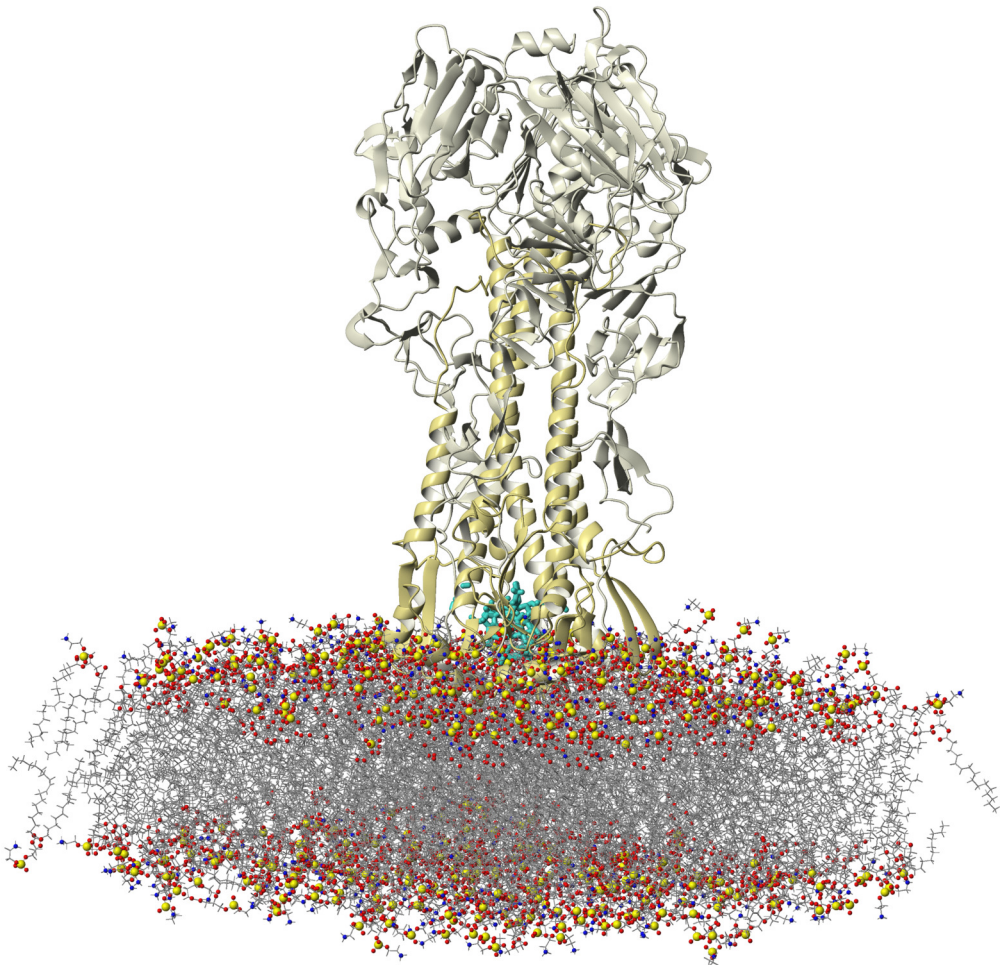

Supplement: Supplementary file 1 [file membranes-11-00757-s001.zip › SupportingMaterials/pae_ha3_orn-eps-converted-to.pdf]

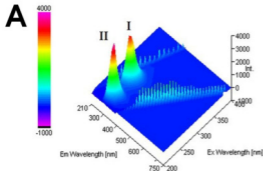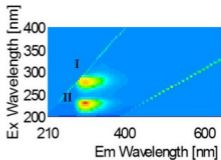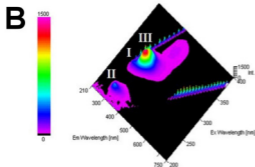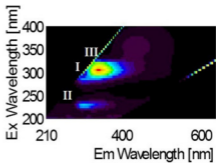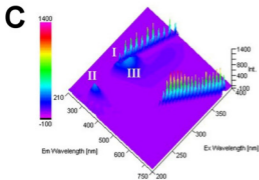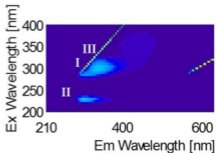

Supplement: Supplementary file 1 [file membranes-11-00757-s001.zip › SupportingMaterials/total_fluorescence_Supp-eps-converted-to.pdf]
